# Electrically tunable THz graphene metasurface wave retarders

**DOI:** 10.1515/nanoph-2022-0812

**Published:** 2023-03-27

**Authors:** Hyunwoo Park, Sodam Jeong, Changwon Seo, Hyeongi Park, Donghak Oh, Jae-Eon Shim, Jaeyeong Lee, Taewoo Ha, Hyeon-Don Kim, Soojeong Baek, Bumki Min, Teun-Teun Kim

**Affiliations:** Department of Physics, University of Ulsan, Ulsan 44610, Republic of Korea; Department of Physics and Energy Harvest-Storage Research Center, University of Ulsan, Ulsan 44610, Republic of Korea; Department of Mechanical Engineering, Korea Advanced Institute of Science and Technology (KAIST), Daejeon 34141, Republic of Korea; Center for Integrated Nanostructure Physics (CINAP), Institute for Basic Science (IBS), Suwon 16419, Republic of Korea; Department of Nano-Mechanics, Nano-Convergence Manufacturing Systems Research Division, Korea Institute of Machinery & Materials (KIMM), Daejeon 34103, Republic of Korea; Department of Physics, Korea Advanced Institute of Science and Technology (KAIST), Daejeon 34141, Republic of Korea

**Keywords:** active polarization control, electrically tunable quarter-wave plate, graphene, graphene metasurfaces, metasurfaces

## Abstract

Anisotropic materials with chirality or birefringence can be used to manipulate the polarization states of electromagnetic waves. However, the comparatively low anisotropy of natural materials hinders the miniaturization of optical components and devices at terahertz frequencies. In this study, we experimentally demonstrate that the relative phase retardation of a THz wave can be electrically controlled by integrating patterned mono- and bilayer graphene onto an otherwise isotropic metasurface. Specifically, we show that a refractive index for one of the orthogonal polarization states can be electrically controlled by modulating graphene’s conductivity, thereby weakening the capacitive coupling between adjacent meta-atoms in an anisotropic manner. With monolayer graphene, phase retardation of 15° to 81° between two orthogonal polarization states can be achieved. Maximum phase retardation of 90° through a metasurface with bilayer graphene suggests its use as a tunable quarter-wave plate. Continuous control from linear- to circular-polarization states may provide a wide range of opportunities for the development of compact THz polarization devices and polarization-sensitive THz technology.

## 1 Introduction

The polarization of electromagnetic waves plays an essential role in a wide range of fields because the electromagnetic responses of materials and devices typically depend on the polarization states of the incident electromagnetic waves. Sophisticated manipulation of polarization states is critical for the organization of complicated optical systems. Birefringence—distinct refractive indices along orthogonal principal axes—has been widely employed to manipulate the polarization states of electromagnetic waves [1, 2]. However, naturally occurring birefringence is extremely weak and requires a substantially long propagation length to obtain adequate phase retardation. Particularly in the THz regime, the lack of natural materials with strong birefringence is an obstacle to the realization of practical polarization components or devices.

For practical applications at THz frequencies, metasurfaces (the two-dimensional counterpart of metamaterials, composed of a two-dimensional array of planar structures) are among the most promising platforms due to the versatility of their design [3–8]. Metasurfaces have significantly improved the performance of conventional THz devices by enabling further miniaturization and tunability [9–15]. For the realization of active metasurfaces, it becomes necessary to incorporate a tunable medium, of which the optical properties can be modulated in real-time under external stimuli. Recently, active THz polarization modulators has been demonstrated with different active medium, such as by using liquid crystal or vanadium dioxide VO_2_ [16, 17]. Among various tunable media, graphene is considered a versatile platform because it exhibits gate-controllable light-matter interactions through the Fermi level shift. Particularly in the THz range, large continuous modulation can be achieved by electrically tuning the joint density of states available for intraband transitions [18–22]. Although significant efforts have been devoted to designing graphene-assisted metasurfaces [23–31], to the best of our knowledge, no experimental result has been reported in the THz range for the realization of tunable quarter waveplates with a full circular polarization state by employing graphene metasurfaces. In Table 1, we summarized the studies for polarization modulation performance.

**Table 1:** Research results on active modulation of ellipticity of polarization at terahertz frequencies.

Modulation source	Measurement type	Active medium	Modulation range	Ref.
Electric/magnetic field	Transmission	Liquid crystal	0.2–1.0 (ellipticity)	[16]
			−0.5 to −1.0 (ellipticity)	
Temperature	Transmission	Vanadium dioxide	−0.72 to −0.99 (ellipticity)	[17]
Electric field	Transmission	Graphene	−0.47 to −0.78 (ellipticity)	[28]
Electric field	Transmission	Graphene	0.15–0.15 (ellipticity)	[29]
Electric field	Reflection	Graphene	3–23 dB (extinction ratio)	[30]

Here, we describe electrically controlled graphene metasurfaces (GMs) that can preferentially modulate the polarization states of THz waves. An active metasurface is formed by integrating mono- or bilayer of graphene micro-ribbons with isotropic metasurfaces. The effective refractive index along one axis can be efficiently modulated by varying the optical conductivity of the graphene micro-ribbons, thereby weakening the capacitive coupling between adjacent meta-atoms in an anisotropic manner. As a result, the polarization states of an incident linearly polarized THz wave can be efficiently changed to a circularly polarized state at the output. This approach will provide a facile way of constructing ultra-compact active polarization modulators and imaging devices.

## 2 Results and discussion

### 2.1 Unit cell design

**Figure 1:**
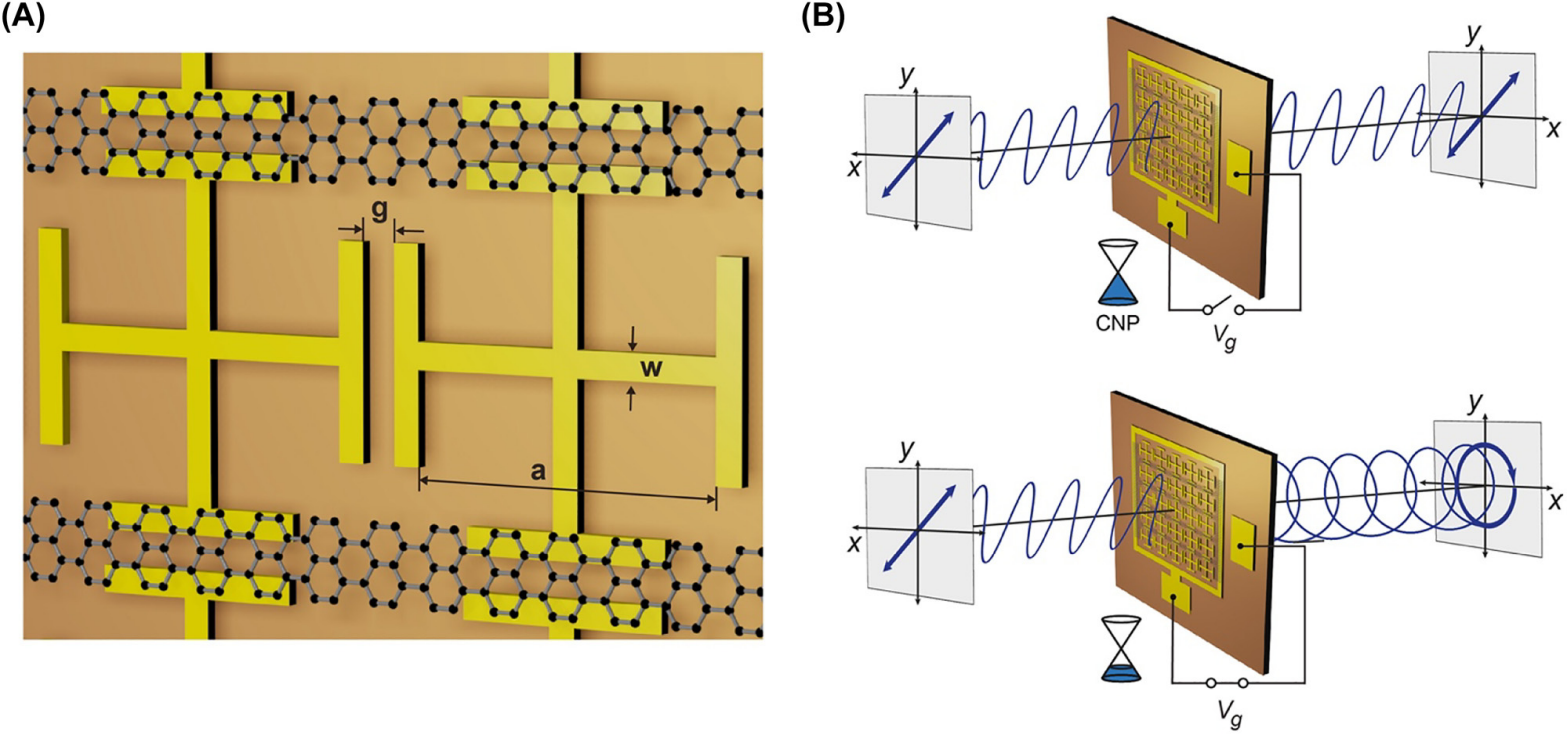
Graphene metasurface for the electrical control of polarization states. (A) Schematic illustration of a graphene metasurfaces composed of double H-shaped meta-atoms (DHMs) and graphene micro-ribbons. Geometric parameters of the fabricated sample are *g* = 2 μm, *w* = 2 μm, and *a* = 24 μm. (B) Polarization states of the transmitted wave at two different Fermi levels of graphene, (top) at the charge neutral point (CNP), and (bottom) at *V*_g,max_.

We first consider isotropic metasurfaces, where each H-shaped meta-atom is orthogonally overlapping with the other (termed in this work as double H-shaped meta-atoms (DHMs)) as schematically illustrated in Figure 1A [4, 32]. The DHMs with small gap widths are characterized by strong capacitive coupling between the adjacent meta-atoms along the *x* and *y* axes, leading to high effective refractive index and correspondingly a large phase shift. Graphene micro-ribbons are attached at the gap between the H-shaped meta-atoms arranged in the *y* -axis, enabling the preferential control of capacitive coupling along that direction. As the optical conductivity of graphene micro-ribbons is efficiently controlled by the gate voltage (*V*_g_, replace by the Fermi level, *E*_F_, in simulations), the *y*-polarized component of incident THz waves can be changed at the output. The gate-controlled anisotropy of the proposed metasurface can be modeled phenomenologically within the Jones matrix formalism as,

$$T=\left[\begin{array}{cc} t_{x}{\text{e}}^{j\phi_{x}} & 0\\ 0 & t_{y}{\text{e}}^{j\phi_{y}\left(V_{g}\right)} \end{array}\right]$$

where *t*_*i*_ and *ϕ* are the amplitude and phase of the complex co-polarized transmission for the *i*-polarized wave (*i* = *x*, *y*), respectively. Only the *y* component of the phase is assumed to be a function of *V*_g_, which can be justified at the operating frequency described below. Graphene exhibits minimal conductivity when *E*_F_ is tuned at the charge neutral point (CNP), where the density of states should vanish in an ideal situation. Because the proposed metasurfaces remain almost isotropic at the CNP (Figure 1B, top), a linearly polarized incident wave maintains its original polarization state at the output. As *E*_F_ increases, the degree of anisotropy is enhanced due to the weakened capacitive coupling resulting from the conductive graphene channel at the gap between the H-shaped meta-atoms arranged in the *y*-axis. When the phase retardation, $\Delta \phi =\phi_{x}-\phi_{y}\left(V_{\text{g}}\right)$ , is made equal to 90° and the specific condition, $\left|t_{x}\right|=\left|t_{y}\right|$ , is simultaneously satisfied, a linearly polarized incident wave can be changed to a circularly polarized transmitted wave, suggesting the potential operation as a tunable THz quarter-waveplate (QWP) (Figure 1B, bottom).

### 2.2 Electrically tunable anisotropy of graphene metasurfaces

The characteristics of GMs are first investigated numerically using a commercial finite element method solver of the CST microwave studio. The geometrical parameters of the metasurface unit-cell used in the simulations are *a* = 24 μm, *w* = 2 μm, and *g* = 2 μm (Figure 1A). The width of the graphene micro-ribbon is 6 μm. For the frequency range of interest, the dielectric constant for gold is tabulated in [33] and can be fitted by using the Drude model with a plasma frequency (*ω*_p_) of 1.37 × 10^16^ rad/s and a collision frequency ( $\left.\gamma \right)$ of 4.07 × 10^13^ rad/s. The complex index of silicon substrate is extracted experimentally by measuring the transmission of a THz wave through the substrate. The optical conductivity of graphene is modelled by the Kubo formula [34], $\sigma \left(\omega \right)=\sigma_{\text{intra}}\left(\omega \right)+\sigma_{\text{inter}}\left(\omega \right)$ , where the intraband transitions are dominant in THz frequency. The formulae for complex conductivity of intraband transitions for graphene are given as follows,

$$\begin{array}{ll} \sigma_{\text{intra}}\left(\omega \right) & =\frac{e^{2}}{\pi \hbar^{2}}\frac{i}{\omega +i\tau^{-1}}\int_{\Delta }^{\infty }d\varepsilon \left(1+\frac{\Delta^{2}}{\varepsilon^{2}}\right)\\ & \;\times \left[f\left(\varepsilon -E_{\text{F}}\right)+f\left(\varepsilon +E_{\text{F}}\right)\right] \end{array}$$

**Figure 2:**
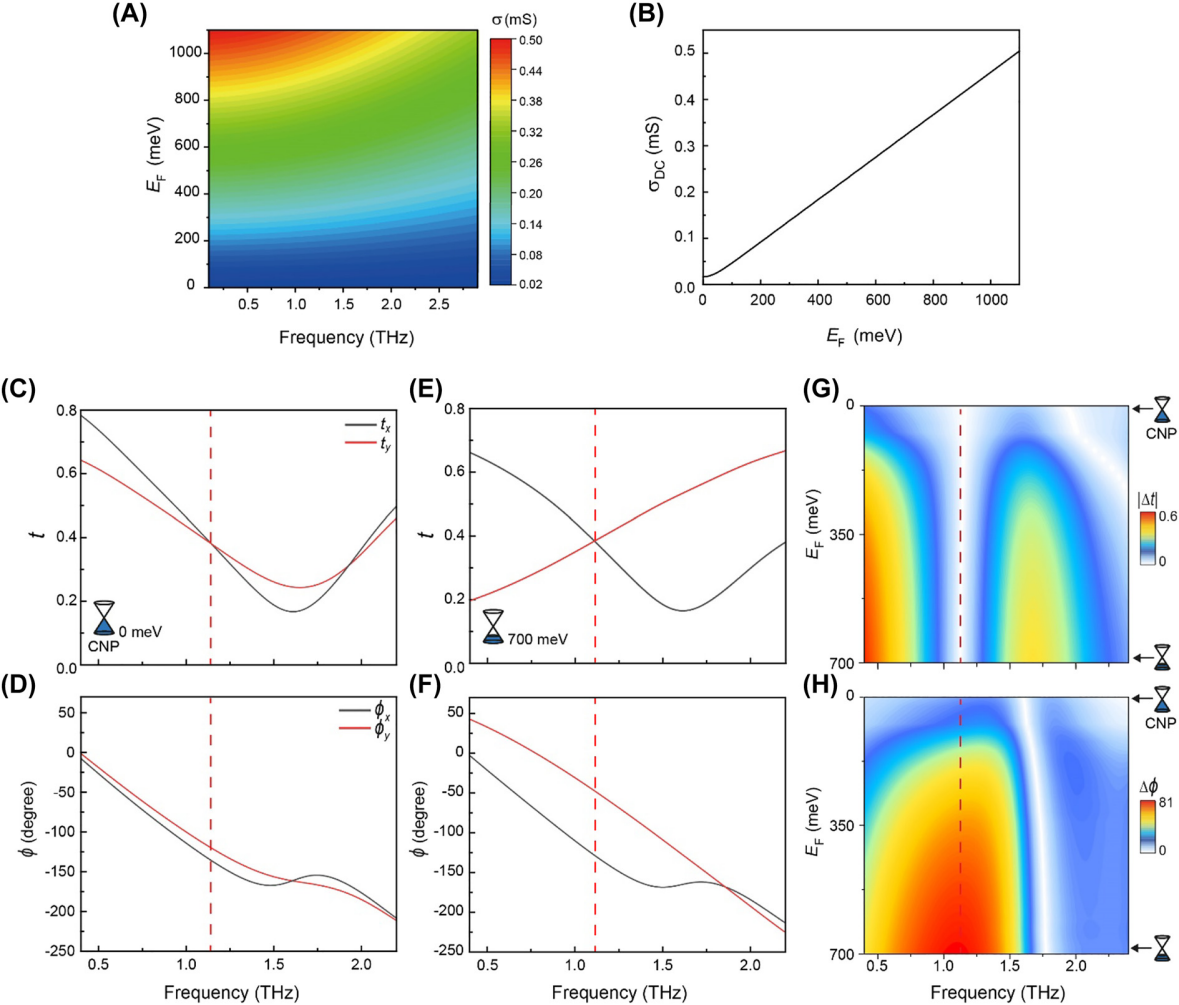
Numerically calculated transmission amplitudes and phases with a variation in the Fermi level. (A) Frequency dependent optical conductivity and (B) DC conductivity as a function of Fermi levels *E*_F_. *τ* is assumed to be 39 fs. (C–F) Simulation results of transmission amplitudes *t*_*x*_, *t*_*y*_, and phases *ϕ*_*x*_, *ϕ*_*y*_ of *x*-(drawn with a black solid line) and *y*-(drawn with a red solid line) polarized waves for the Fermi levels of *E*_F,CNP_ = 0 meV and *E*_F, max_ = 700 meV, respectively. The dashed red line corresponds to the frequency, at which the linear dichroism becomes zero while the relative phase retardation becomes maximized. (E, F) Differences in transmission amplitudes |Δ*t*| and phase retardations Δ*ϕ* mapped as a function of the Fermi level and the frequency.

Here, *e*, *τ* and ∆ are an electron charge, an intraband relaxation time and a half bandgap energy from the tight-binding Hamiltonian near *K*-points of the Brillouin zone, and *f*(*ɛ* − *E*_F_) is the Fermi distribution function with Fermi level *E*_F_. Figure 2A and B shows the real value of optical conductivity and direct current (DC) conductivity as a function of *E*_F_. The plots are obtained by assuming the thickness of graphene is 1 nm and the intraband scattering *τ* = 39 fs. Figure 2C–F shows simulated transmission amplitudes *t*_*x*_, *t*_*y*_ and phases *ϕ*_*x*_, *ϕ*_*y*_ of *x*- and *y*-polarized THz waves through the GMs with the Fermi levels of *E*_F,CNP_ = 0 meV (Figure 2C and D) and *E*_F, max_ = 700 meV (Figure 2E and F). Although the Fermi level of the graphene is set near the CNP, slight discrepancies are still observable in the transmission amplitudes (Figure 2C) and phases (Figure 2D) of the two orthogonal polarizations; these differences are attributed to the intrinsic lower conductivity limit of graphene at CNP [35, 36]. The resonance frequency of the GM for the *y*-polarized incident wave is found to redshift with an increase in *E*_F_ and correspondingly in the conductivity of graphene [37]. Still, it should be noted that the transmission spectra are almost invariant for the *x*-polarized wave. To determine the operating frequency, at which the linear dichroism becomes zero while the relative phase retardation becomes maximized, the difference in transmission amplitudes, $\left|\Delta t\right|=|t_{x}-t_{y}|$ , and the phase retardation, Δ*ϕ*, are plotted as a function of *E*_F_ in Figure 2G and H. At the frequency of 1.10 THz (denoted by a red dashed line), one can see that Δ*ϕ* can be continuously modulated while $\left|\Delta t\right|$ remains almost zero. The maximum phase retardation Δ*ϕ* = 81° is achieved at the Fermi level of *E*_F_ = 700 meV. These simulation results clearly show that the polarization state of a transmitted THz wave can be efficiently and continuously modulated by changing the optical conductivity of graphene micro-ribbons.

### 2.3 Fabrication and characterization of GMs

**Figure 3:**
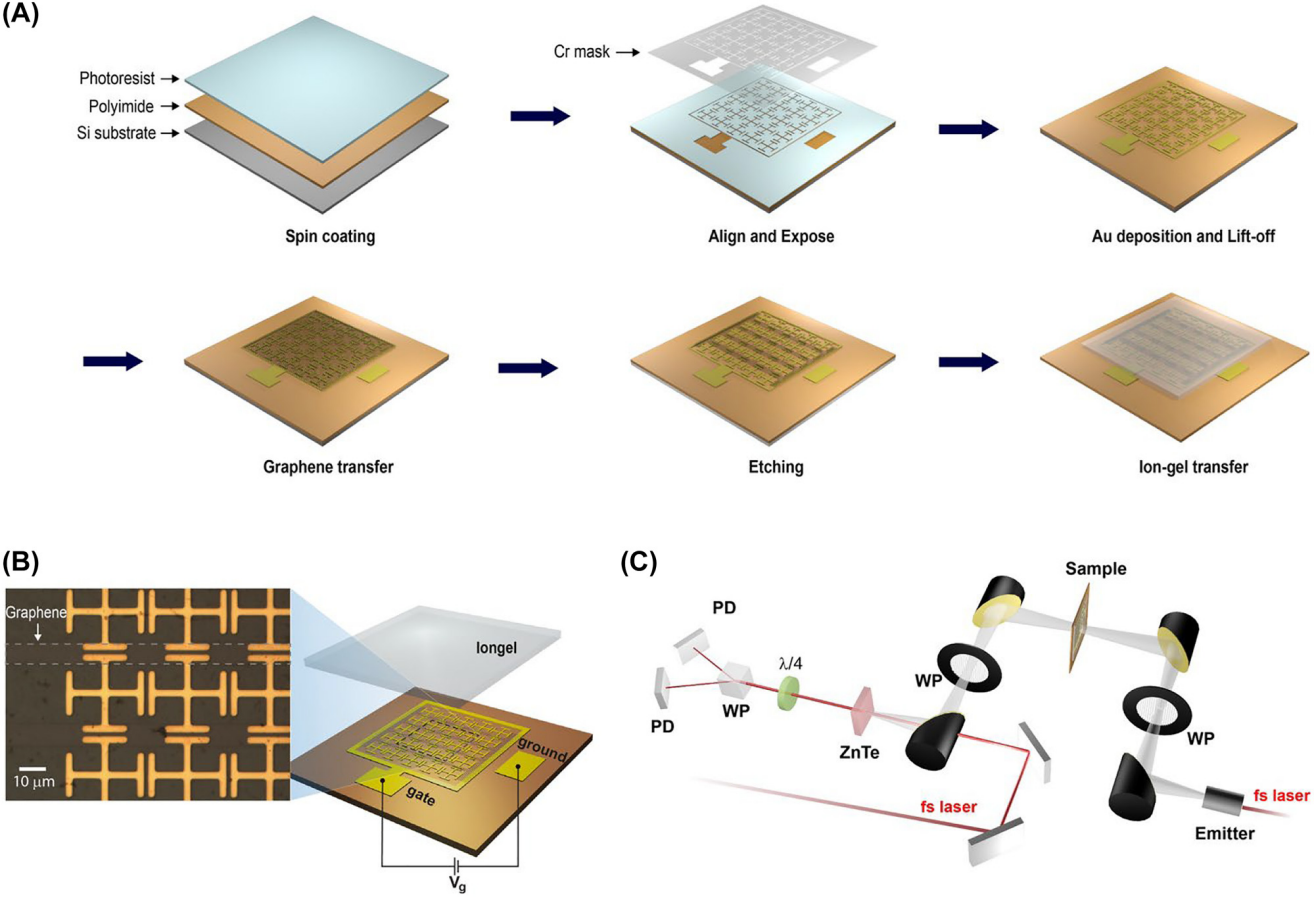
Schematic illustration of fabrication process, artistic rendering and microscopic images of GMs, and THz-TDS setup in detail. (A) Schematic representation of GM preparation steps. (B) Schematic rendering of the GMs. inset: Microscopic image of the fabricated GM. (C) Schematic illustration of the THz-TDS setup.

The GM is fabricated using standard microelectromechanical system (MEMS) technology (Figure 3A). A polyimide solution (PI-2610, HD MicroSystems) is spin-coated onto a silicon wafer with a target thickness of 1 μm. The metallic meta-atom structures are made of 100 nm-thick gold and attached to the substrate with a 20 nm-thick chromium adhesive layer. To bridge the capacitive gaps between DHMs with graphene micro-ribbons, CVD-grown graphene is transferred to the substrate with previously patterned DHMs. For the graphene transfer, PMMA (poly(methyl methacrylate), C2, Microchem) is used as a supporting layer. The transferred large-area graphene is then patterned by ultraviolet (UV) lithography. After UV exposure and development of bilayer photoresist (PMGI and HKT 501), the portions of graphene not covered by the resist are etched using a plasma asher. The use of two types of photoresists during the fabrication of graphene micro-ribbons serves a specific purpose. After developing the top layer photoresist (HKT 501), an oxygen plasma is employed for 5 min with a power value of 50 W to create a ribbon-patterned graphene according to the shape of the developed photoresist. However, the top layer of photoresist can harden during this process due to the plasma-induced increase in temperature, which may complicate further steps. Therefore, a second layer of photoresist (PMGI) is added beneath the top layer to shield it from plasma exposure but is ultimately removed. This underlying layer of photoresist not only prevents hardening but also enhances the ease of removing the photoresist. As a final step, GMs were peeled-off from the silicon substrate. The conductivity of graphene micro-ribbon is made controllable by applying a voltage between the square ring–shaped gate electrode and the ground electrode, both of which are patterned on polyimide films. Two ends of the graphene micro-ribbon are attached to the square ring-shaped gate electrode. The ion-gel layer, incorporated here for low voltage operation of GMs, encapsulates all the electrodes as well as the graphene micro-ribbons (Figure 3B). The prepared GMs are then characterized by THz time-domain spectroscopy (THz-TDS), which provides information on the amplitude and phase of the transmitted THz wave (Figure 3C). The main parts of the system consist of a Ti-sapphire femtosecond laser (Mai-Tai, Spectra-physics, with a central wavelength of 800 nm) operating at a repetition rate of 80 MHz, a photoconductive antenna (iPCA, BATOP) for the generation of a THz wave, and a 1 mm-thick zinc telluride crystal for the detection. The spectral range of the produced THz signal extends from 0.1 to 2.5 THz. Two wire-grid THz polarizers were used to increase the precision of polarization detection.

### 2.4 Experimental results

**Figure 4:**
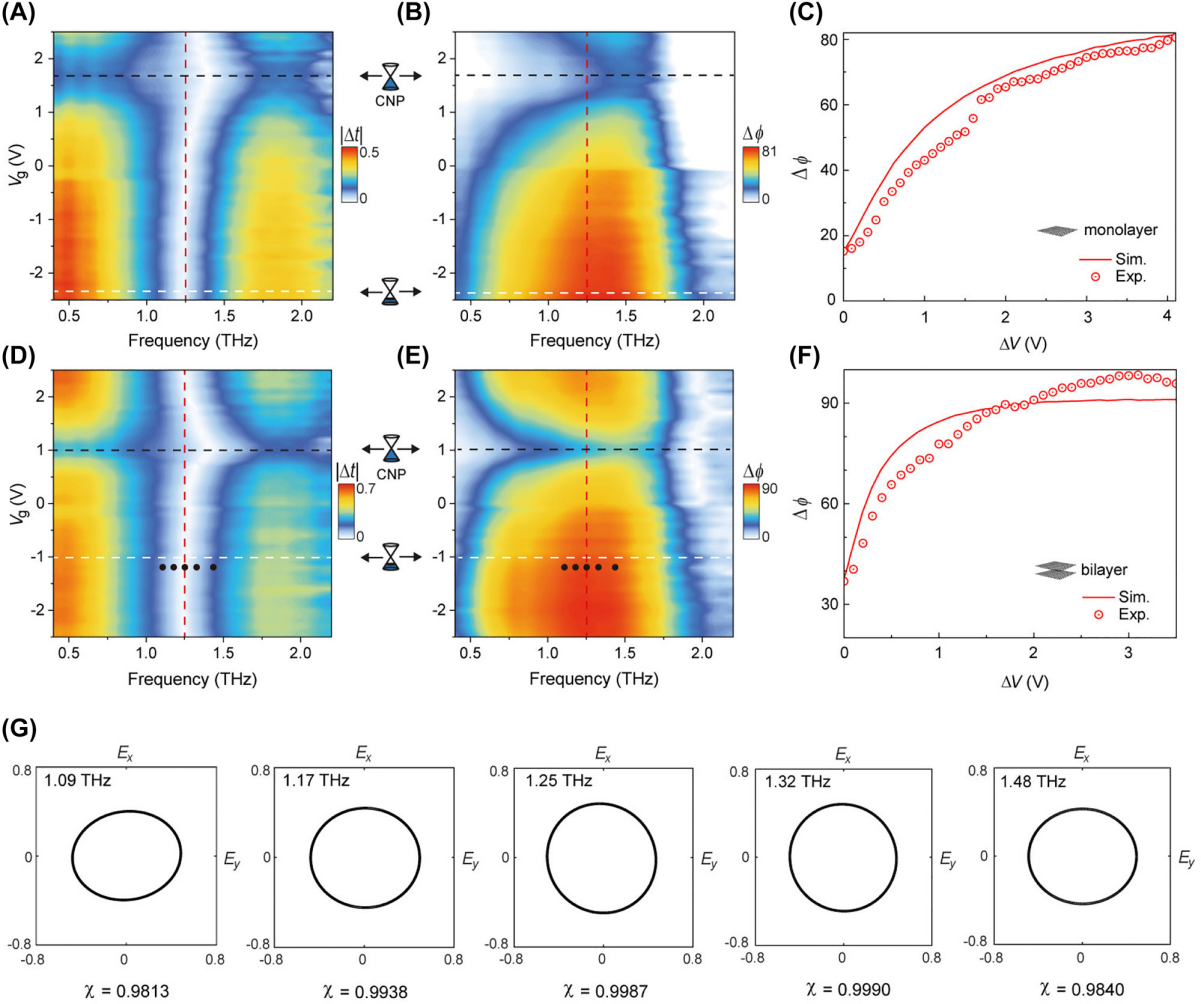
Experimental results of active polarization state control. Experimentally measured $\left|\Delta t\right|$ and Δ*ϕ* of the GMs fabricated with (A, B) mono- and (D, E) bilayer graphene micro-ribbons. These maps are plotted as a function of frequency and gate voltage. The red, black, and white dashed lines represent $f_{\left|\Delta t\right|\approx 0}$ , *V*_CNP_, and *V*_Δ*ϕ*, max_, respectively. Measured (red dot circle) and simulated (red solid line) Δ*ϕ* of (C) the mono- and (F) the bilayer GMs plotted as a function of Δ*V*. In both plots, the frequency is set to 1.25 THz. (G) Polarization states at different frequencies with ellipticity *χ* ≥ 0.98 at *V*_g_ = −1.2 V (black dots).

First, we start by characterizing the monolayer graphene-based GMs. The measured frequency-dependent values of $\left|\Delta t\right|$ and Δ*ϕ* as a function of gate voltage are shown in Figure 4A and B. By inspecting the measured trace of transmission difference minima, the gate voltage corresponding to the CNP, *V*_CNP_, is estimated to be approximately 1.6 V (drawn with a black dashed line in Figure 4A and B). At the frequency of $f_{\left|\Delta t\right|\approx 0}$ = 1.25 THz, the transmission difference $\left|\Delta t\right|$ becomes almost zero regardless of the variation in gate voltage; furthermore, at this specific frequency, it can be noted that the relative phase modulation Δ*ϕ* exhibits the largest change with the variation in gate voltage (denoted by a red dashed line in Figure 4A and B). A minimum Δ*ϕ* at 1.25 THz was measured to be 15°. This non-zero phase difference is due to the residual carrier doping, which resulting in a non-zero minimum conductivity value [38]. In Figure 4C, the measured relative phase retardation is plotted as a function of the gate voltage referenced to that of the CNP, $\Delta V=\left|V_{\text{g}}-V_{\text{CNP}}\right|$ , and compared with the simulation results (drawn with a red line in Figure 4C). At Δ*V* = 4.1 V, the maximum relative phase retardation Δ*ϕ*_max_ is measured to be 81°, the value of which falls a little short of a quarter-wave retardation. This is at tributed to the limited controllability of monolayer graphene conductivity, which is in part caused by the relatively short intraband scattering time *τ* [37]. To increase the conductivity of channels bridging the gap between the DHMs, we fabricated GMs patterned with bilayer graphene, which is more conductive than monolayer graphene [39, 40]. Figure 4D and E shows that *V*_CNP_ is located at 1.0 V, while a minimum $\left|\Delta t\right|$ is observed at 1.25 THz, as is the case with monolayer GMs. Fitting the measured relative phase retardation curve to the estimated curve (Figure 4F) reveals that the initial Fermi level has risen to 45 meV, which validates our bilayer approach. At 1.25 THz, the minimum Δ*ϕ* is measured to be 37°, while the quarter-wave retardation (Δ*ϕ* = 90°) is observed at a comparatively low gate voltage of *V*_g_ = −1.0 V (denoted by white dashed lines in Figure 4D and E).

**Figure 5:**
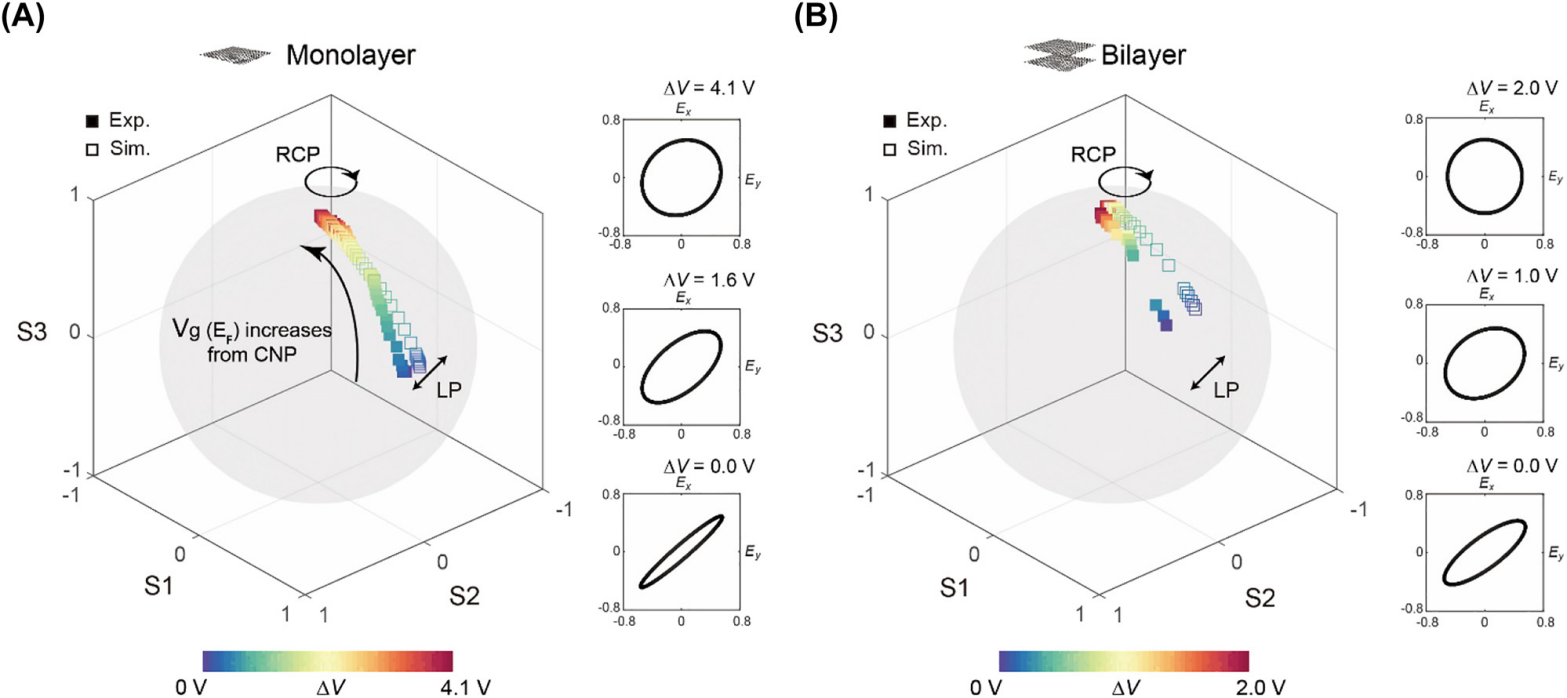
Evolution of polarization states represented on a Poincaré sphere. (A, B) Experimentally extracted (squares) and numerically simulated (hollow squares) polarization states parametrically plotted on a Poincaré sphere for GMs fabricated with (A) mono- and (B) bilayer graphene. Inset: experimentally extracted polarization ellipse at three different values of Δ*V*.

To gain a better understanding of the evolution of polarization states, a Poincaré sphere representation is used to visualize numerically calculated and experimentally extracted polarization states as a function of V (Figure 5). Here, the Stokes parameters are given by [41],

$$\;S_{0}=t_{x}^{2}+t_{y}^{2}=1$$

$$\;S_{1}=t_{x}^{2}-t_{y}^{2}=\cos2\psi$$

$$\;S_{2}=2t_{x}t_{y}\cos\Delta \phi =\sin2\psi \cos\Delta \phi$$

$$\;S_{3}=2t_{x}t_{y}\sin\Delta \phi =\sin2\psi \;\sin\Delta \phi$$

where the angle *ψ* characterizes the ratio of transmission amplitudes (tan *ψ* = *t*_*x*_/*t*_*y*_). It is worth noting that the polarization state is solely defined by Δ*ϕ*, which is in turn determined by |Δ*V*|, at the frequency of $f_{\left|\Delta t\right|\approx 0}$ , where *ψ* is equal to 45°. The transmitted wave becomes elliptically polarized due to the residual conductivity of graphene at the CNP (Δ*V* = 0 V), which results in an ellipticity (*χ* = *S*_3_/*S*_0_) of 0.29 for monolayer GMs and 0.60 for GMs with bilayer graphene. For GMs with monolayer graphene, the ellipticity can be raised up to 0.98 with an increase in gate voltage, but a fully circular polarization state cannot be reached (*χ* = 1) (Figure 5A). In contrast, for GMs with bilayer graphene, it is shown in Figure 5B that a full circular polarization state is accessed at Δ*V* = 2.0 V (Figure 5B). While we focus on realizing the perfect circular polarization with *χ* = 1, the frequency range with Δ*ϕ* ≈ 90° and *χ* ≥ 0.98 in the GMs with bilayer graphene is more than 390 GHz which exhibit broadband polarization modulation as shown in Figure 4G.

**Figure 6:**
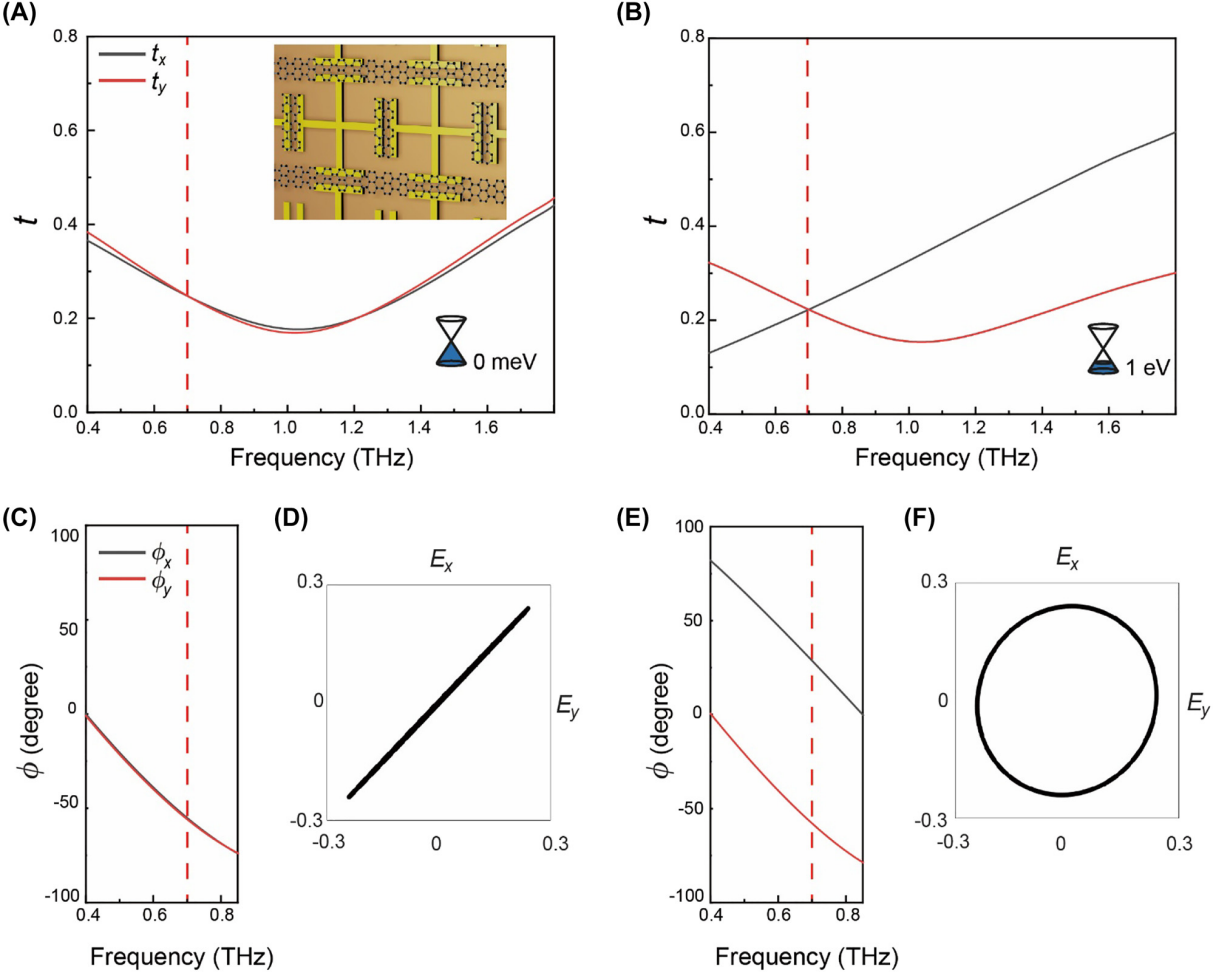
Numerically calculated full phase retardation from linear to circular polarization. Calculated (A, B) transmission amplitude *t*_*x*_, *t*_*y*_ and (C, E) phases *ϕ*_*x*_, *ϕ*_*y*_ of *x*- and *y*-polarized waves through the GMs with *x*-axis gap covered by cut bilayer graphene micro-ribbons for the Fermi levels of *E*_F_ = 0 meV and *E*_F_ = 1 eV, respectively. (D, F) Polarization states for 0 meV and 1 eV at 0.70 THz.

However, it is important to consider that the achieved result is due to an increase in the residual conductivity of bilayer graphene at the CNP, indicated by an initial ellipticity value of 0.60. To address this issue, DHMs can be designed with smaller gap widths and by covering the gap with additional cut graphene arranged in the *y*-axis. This approach can result in a higher refractive index due to strong capacitive coupling [4]. Figure 6 shows simulated *t*_*x*_, *t*_*y*_ (Figure 6A and B) and phases *ϕ*_*x*_, *ϕ*_*y*_ (Figure 6C and E) of THz waves through the GMs with a gap width of 500 nm. Additional cut bilayer graphene micro-ribbons are attached at the gap between the meta-atoms arranged in the *x*-axis. To minimize the residual conductivity, we assume *τ* = 16 fs. The simulation results show that the phase retardation can be fully covered from linear polarization (*χ* = 0.013 for *E*_F_ = 0 meV) (Figure 6D) to circular polarization (*χ* = 0.998 *E*_F, max_ = 1 eV) (Figure 6F) at 0.70 THz, which can be utilized as a full range active quarter wave plate.

## 3 Conclusions

In this work, we demonstrate that the anisotropy of GMs can be changed electrically, resulting in a significant change in the relative phase retardation of an incident THz wave. The anisotropic resonance weakening of the GM is enabled by controlling the optical conductivity of constituting graphene micro-ribbons. Furthermore, we numerically demonstrated that a full range phase retardation modulation by reducing the gap size of GM and covering the gap with additional cut graphene arranged in the *y*-axis. Benefiting from the continuous and accurate control of polarization states, the proposed GM platform may become a building block for the realization of compact (whole device thickness *d*_sample_ = 11 μm <21/λ ) and tunable THz polarization devices.
